# Galectin-3 administration drives remyelination after hypoxic-ischemic induced perinatal white matter injury

**DOI:** 10.3389/fncel.2022.976002

**Published:** 2022-09-20

**Authors:** Qian Wang, Sihao Diao, Han Qiu, Ruiwei Gao, Minjie Wang, Qiufan Chen, Mili Xiao, Zhihua Li, Chao Chen

**Affiliations:** ^1^Department of Neonatology, Children's Hospital of Fudan University, Shanghai, China; ^2^Key Laboratory of Neonatal Diseases, National Health Commission, Shanghai, China; ^3^Department of Neonatology, Women and Children's Medical Center of Guangzhou, Guangzhou, China

**Keywords:** galectin-3, oligodendrocytes, myelin formation, perinatal white matter injury, M2 microglia

## Abstract

Hypoxic-ischemic (HI) induced perinatal white matter injury (PWMI) is a major cause of neurologic disabilities characterized by selective oligodendroglial death and myelin disruption. Galectin-3 (Gal-3) modulates postnatal subventricular zone gliogenesis and attenuates ischemic injury. However, the association between Gal-3 and myelin formation still remains unclear. In this study, we first perform Gal-3 knockdown (KD) to identify the importance of Gal-3 on myelin formation. Our results show impeded myelin formation, manifested by Olig2/CC1 (+) mature oligodendrocytes number, expression of oligodendroglial maturation-associated markers (MBP and CNPase), and myelin thickness and integrity. Then we perform recombinant Gal-3 (rGal-3) administration by intracerebroventricular injection. Notably, although rGal-3 administration shows no beneficial effect on oligodendrogenesis and myelin formation under normal condition, our results show that rGal-3 administration attenuates cognitive deficits and drives remyelination after PWMI, which are coupled to signs of enhanced myelin resiliency and cognition. Also, our results indicates that the significant increases in substrates for remyelination of rGal-3 administration are accompanied by enhanced Iba-1 (microglia marker)/ Mrc1 (M2 marker) (+) microglia and decreased Iba-1/ iNOS (M1 marker) (+) microglia. Altogether, our data in this research confirm the association between Gal-3 and myelin formation, underscore its position for the capacity for remyelination and restoration of function, and unveils the efficacy of rGal-3 administration with anti-inflammatory phenotype microglia (M2 microglia) activation. Thus, the findings suggest that Gal-3 plays a significant role in myelin formation and remyelination restoration.

## Introduction

Pathophysiological changes of white matter injury (WMI) involve selective oligodendrocyte death, myelin disruption, and pathological glial cell activation (Back, 2017). The feature that distinguishes WMI from other neurological disorders is the formation of confluent demyelinated plaques. Myelin is the multilayer lipid membrane that wraps and insulates axons, which provides insulation for axons and normalizes function of nervous system (Sun et al., 2018; Elbaz and Popko, 2019).

Throughout neurodevelopment, differentiation of oligodendrocyte linage is precisely controlled by extracellular and intrinsic signals (Sun et al., 2018; Elbaz and Popko, 2019). Galectin-3 (Gal-3), which is a carbohydrate-binding protein belonging to the lectin family (Dong et al., 2018; Wang et al., 2019). Gal-3 accelerates cell proliferation and differentiation, regulates cell-cell interactions within the extracellular matrix, modulates postnatal subventricular gliogenesis, and plays pleiotropic roles in central nervous system (CNS) (Hoyos et al., 2014; Chip et al., 2017; Dong et al., 2018; Kariya et al., 2018; Al-Dalahmah et al., 2020). Gal-3 deletion downregulates nerve growth factor (NGF) and brain derived neurotrophic factor (BDNF), suppresses microglial proliferation and activation, and causes anxiogenic effect in wild type healthy mature animal (Lalancette-Hébert et al., 2012; Stajic et al., 2019). Gal-3 deletion also decreases ischemia-induced angiogenesis, inhibits the proliferation of neural progenitors, enlarges ischemic lesion, and increases the number of apoptotic neurons after CNS injury (Lalancette-Hébert et al., 2012; Chip et al., 2017). However, researches have unveiled the association between Gal-3 and myelin formation (Pasquini et al., 2011; Thomas and Pasquini, 2019, 2020). In this study, we first performed Gal-3 knockdown by adeno-associated virus (AAV) and confirmed the importance of Gal-3 on myelin formation, presented by Olig2/CC1 (+) mature oligodendrocyte number, expression of myelin associated markers (MBP and CNPase), and myelin thickness and integrity.

Trophic factors such as BDNF, tissue inhibitor of metalloproteinase-1 (TIMP-1), and leukemia inhibitory factor (LIF) administration constructively facilitate a favorable environment for tissue repair and emerge as viable repair therapies in CNS injury (Dougherty et al., 2000; Fulmer et al., 2014; Jiang et al., 2016; Ohtomo et al., 2018; Lin et al., 2020). Researches have indicated microglia activation as a hallmark of demyelinated injury (Miron et al., 2013; Ohtomo et al., 2018; Zabala et al., 2018; Elbaz and Popko, 2019; Hughes and Appel, 2020; Li et al., 2020). However, activated microglia are highly heterogeneous immune cells with continuous spectrum activation statuses, which are pro-inflammatory phenotype (M1 microglia) and anti-inflammatory phenotype (M2 microglia) (Miron et al., 2013; Zabala et al., 2018). M2 microglia has been testified not only accelerates tissue repair by releasing neurodevelopmental factors and anti-inflammatory cytokines, but also promotes differentiation of oligodendrocyte by myelin debris phagocytosis (Zabala et al., 2018).

Gal-3 has been demonstrated upregulates after perinatal white matter injury (PWMI) by our previous work and other studies, and Gal-3 has been detected co-stains with Iba-1, a marker of microglia (Chip et al., 2017; Stajic et al., 2019; Wang et al., 2019). Since Gal-3 knockdown causes neurodevelopmental retardation and demyelination, we wonder if exogenously Gal-3 administration is advantageous for myelin formation (Pasquini et al., 2011; Hoyos et al., 2014). Thus, we performed intracerebroventricular injection of recombinant Gal-3 (rGal-3) administration. Differ from the previous researches (Thomas and Pasquini, 2019, 2020), our data demonstrated no improvement of rGal-3 administration on accelerating oligodendroglial differentiation. Notably, we found rGal-3 administration improve remyelination and attenuate cognition after HI induced PWMI, manifested by increased mature oligodendrocytes number, upregulated MBP and CNPase expression, promoted myelin thickness and integrity, and improved spatial learning and memory. Meanwhile, we attempted to explore if rGal-3 administration participates in activated microglia phenotype regulation and modulation of inflammatory cascade. Notably, our results indicate that rGal-3 administration attenuates pro-inflammatory responses and drives microglia activation toward anti-inflammatory phenotype (M2 microglia), which indicates rGal-3 administration participants in activated microglia phenotype regulation and inflammatory responses modulation. Here, our results highlight the importance of Gal-3 on myelin formation and explore the underlying mechanisms on remyelination of rGal-3 administration.

## Materials and methods

### Animals

Sprague–Dawley rats were purchased from Shanghai Sippr-BK laboratory animal Co. Ltd. All animals were group-housed in a temperature-controlled environment with a 12:12-h light /dark cycle and were given access to food and water ad libitum. This research was approved by the Animal Ethics Committee of Fudan University and were carried out in accordance with the guidelines of the National Institutes of Health on animal care and the ethical guidelines.

### Adeno-associated virus and tail vein injection

The sequence used for RNAi targeting *lgals3* (encodes Gal-3) was GGTTGGCGGTCAATGATGTT. AAV that expressed shRNA targeting a non-specific sequence (CGCTGAGTACTTCGAAATGTC) was used as a normal control. AAV9-shLgals3 and AAV9-shNC were designed and purchased from the Shanghai Genechem Co. Ltd, China. Sprague–Dawley rat pups were injected with 100 μl AAV9-shLgals3 or AAV9-shNC from tail vein at 10-day old (0-day old as the day of birth). The injected virus of is approximately 5.6 × 10^11^ V.G./Rat.

### Intracerebroventricular injection

The intracerebroventricular injection was performed as previously described (Deng et al., 2015). Recombinant Gal-3 (R&D Systems, the USA, 2 μl, 5 μg/ml, dissolved in sterile PBS) or sterile PBS (2 μl) were delivered at a rate of 0.4 μl/min.

### Perinatal white matter injury model of Sprague-Dawley rat

Our previous studies have demonstrated that Rice-Vanucci HI operation disrupts myelin formation and causes white matter injury (Huang et al., 2009; Wang et al., 2019). The PWMI model was performed as described preciously (Huang et al., 2009; Deng et al., 2015; Wang et al., 2019). Briefly, P3 (P0 as the day of birth) Sprague–Dawley rats were anesthetized with isoflurane, the right common carotid artery was cut between double ligatures. The surgery never exceeded 5 min and any pups with bleeding or respiratory failure were excluded. After surgery, pups recovered for 1.5 h, then the operator placed them in a container maintained at 37°C. Hypoxia was induced by perfusing the container with humidified 6% oxygen in nitrogen gas mixture for 2.5 h. Sham-operated rat pups were randomly chosen from the same litters of HI rats and had neither common carotid artery ligation nor a period of hypoxia.

### Western blotting

Rats were sacrificed and the right hemisphere of brain was dissected. Protein extraction and western blotting were performed as previously described (Wang et al., 2019). Primary antibodies: Olig2 (1:1,000) (Abcam, the UK), MBP (1:1,000, Biolegend, the USA), CNPase (1:1000) (Cell Signaling Technology, the USA), β-actin (1:1,000) (Absin, China), followed by incubation with HRP-conjugated secondary antibody (1:1,000) (Absin, China). Results were analyzed quantitatively *via* densitometry using ImageJ software (NIH, USA). Data represented the mean ± SEM of three independent experiments.

### Histological examination and immunofluorescence staining

Rats were anesthetized with chloral hydrate and perfused with cold PBS followed by 4% paraformaldehyde (PFA). We performed coronal frozen sections and selected a section approach to mid-brain, hematoxylin-eosin (H-E) staining, and immunofluorescence staining were performed as previously described (Deng et al., 2015; Wang et al., 2019). Primary antibodies: Olig2 (1:200), CC1 (1:200), Mrc1 (1:200), Iba-1 (1:200), iNOs (1:200) (Abcam, the UK); MBP (1:200, Biolegend, the USA); Ki67 (1:100), CNPase (1:400) (Cell Signaling Technology, the USA); Arg1 (1:200, Biolegend, the USA). Primary antibodies were detected with the Alexa Fluor 488 goat anti-mouse (1:500) and Alexa Fluor 647 goat anti-rabbit (1:500) (Abcam, the UK) secondary antibody. Fluorescent signals were visualized using a LeicaTCS-SP8 fluorescence microscope. Results were analyzed quantitatively *via* densitometry by using ImageJ software (NIH, USA).

### Transmission electron microscopy

Rats were anesthetized with chloral hydrate and perfused with cold PBS followed by 4% PFA and 1% glutaraldehyde in 0.1 M phosphate buffer (pH 7.2). After perfusion, brains were dissected out, and the corpus callosum was post-fixed in 4% PFA and 2.5% glutaraldehyde in 0.1 M phosphate buffer (pH 7.2). Transmission electron microscopy was performed as previously described (Deng et al., 2015). We randomly selected images and analyzed by the NIH Image J 1.46 software (National Institutes of Health, USA). G-ratio was calculated by the ratio between the inner axonal and outer total diameter of the myelin sheath of myelinated axons.

### Morris water maze

The Morris water maze was performed as described previously (Wang et al., 2018). Briefly, we recorded the escape latency and swim distance of the navigation test, and platform crossing, time spent and percentages of distance in the target quadrant of the probe trail. Data were collected and analyzed by using the Morris water maze software.

### Statistical analysis

The statistical analyses were performed using Prism 8.0 (GraphPad Software, United States), and continuous variables were presented as the mean ± SEM. Differences between two groups were evaluated by using the 2-sample *t*-test. One way ANOVA followed by Tukey's multiple comparison test or Two-way ANOVA followed by Bonferroni's multiple comparisons test was conducted for comparisons among multiple groups. Survival analysis was performed with a Kaplan-Meier curve and log rank test. We used One-Way ANOVA and a value of *P* < 0.05 was considered statistically significant. Shapiro-Wilk test for normality were used to assess data distribution, and all the statistical analyses passed normality test.

## Results

### Gal-3 knockdown causes developmental retardation

In order to identify the functional importance of Gal-3, we performed Gal-3 knockdown with AAV9-shLgals3 by tail vein injection at 10 day old (0 day old as the day of birth). Western blotting was performed to identify Gal-3 expression in CNS at 10 days after injection (P10). Our data confirmed a significant Gal-3 downregulation of rats in AAV9-shLgals3 group (SF 1A). Body weight were measured at the day of AAV9 injection (P0) and 10 day (P10), 14 day (P14), 21 day (P21), 28 day (P28) and 35 day (P35) after injection. Although no significant difference was observed at P0 and P10, rats in AAV9-shLgals3 group showed decreased body weights at P14/21/28/35 (SF 1B). Rats of each group were sacrificed as planned at P10/14/21/28/35. Unexpectedly, 11 of 49 rats in AAV9-shLgals3 group died within 35 days after injection (SF 1C-D). Notably, a survival curve was presented in SF 1C, although a *P* < 0.05 was not detected, such kind of irregular death was not observed in AAV9-shNC group during the same period (SF 1C-D).

### Gal-3 knockdown impedes myelin formation and causes cognitive deficits

**Figure 1:**
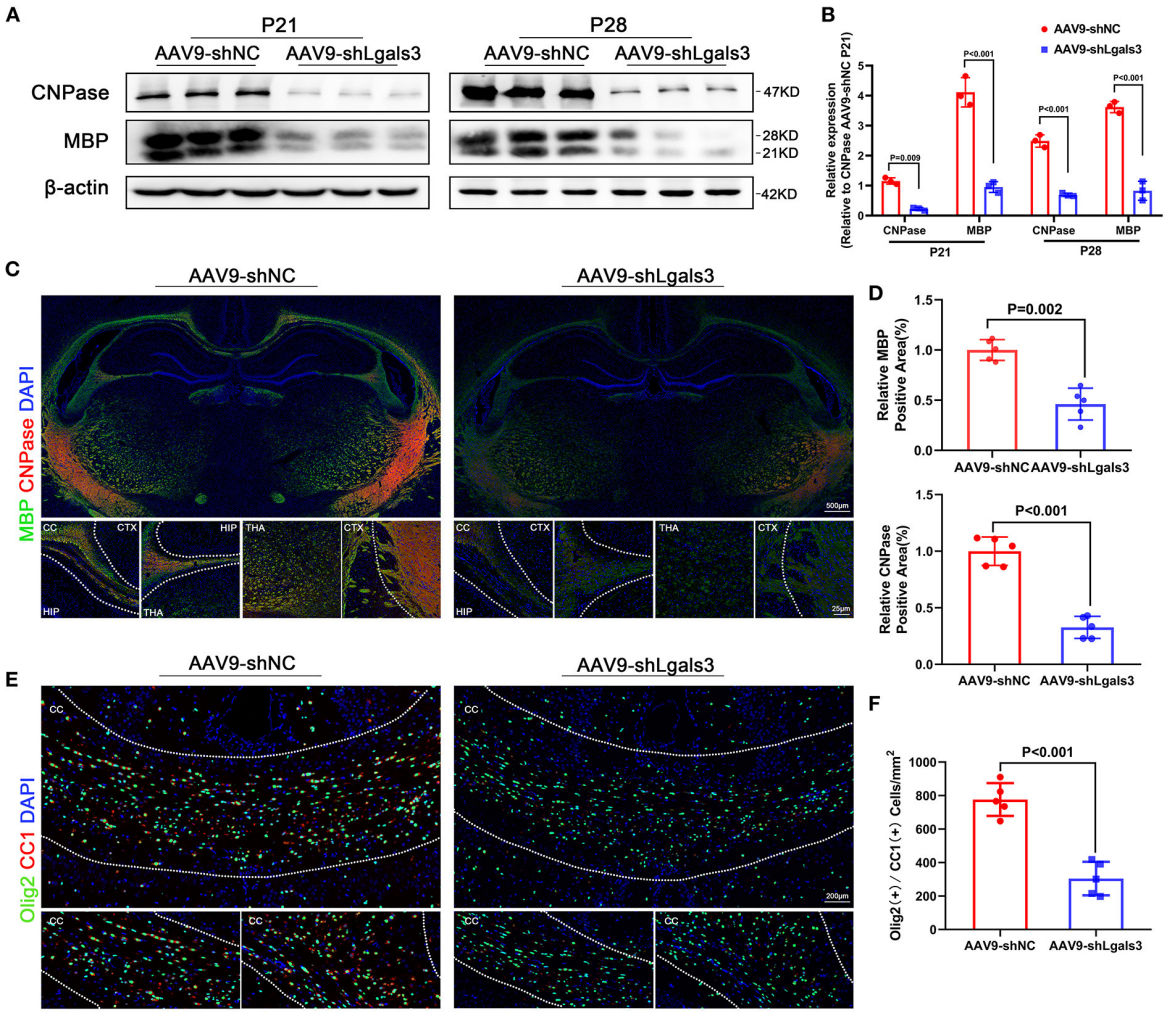
Gal-3 inhibition by AAV9-shLgals3 injection causes decreased mature oligodendrocytes number and oligodendroglial differentiation marker expression. Western blotting images **(A)** and its quantification **(B)** of MBP and CNPase at P21 and P28, *N* = 3. Representative immunofluorescence images **(C)** and its quantification **(D)** of MBP and CNPase at P28, *N* = 5. Representative double stained immunofluorescence images **(E)** of Olig2 / CC1 (+) cells and its quantification **(F)** in the corpus callosum at P28, *N* = 5. CTX, Cortex; CC, Corpus callosum; HIP, Hippocampus; THA, Thalamus.

In order to evaluate the association between Gal-3 and myelin formation, we performed immunofluorescence of Olig2 (an oligodendrocyte linage marker) and double stained Olig2/Ki67 (a marker of proliferation) to identify total cell and the proliferating cell number of oligodendrocyte linage at P21, our results showed no significant difference between AAV9-shNC and AAV9-shLgals3 groups on Olig2 (+) and Olig2/Ki67 (+) cell number in the corpus callosum (SF1E-G). We performed western blotting of MBP and CNPase (oligodendroglial maturation-associated markers) at P21 and P28 and immunofluorescence of MBP and CNPase at P28. Then, we double stained Olig2 (an oligodendrocyte linage marker) with CC1 (a mature oligodendrocyte marker) to identify the number of mature oligodendrocytes. Results of Figures 1A–D showed decreased MBP and CNPase expression in AAV9-shLgals3 group at P21 and P28. Results of Figures 1E,F showed decreased Olig2 / CC1 (+) mature oligodendrocytes in the corpus callosum in AAV9-shLgals3 group at P28.

**Figure 2:**
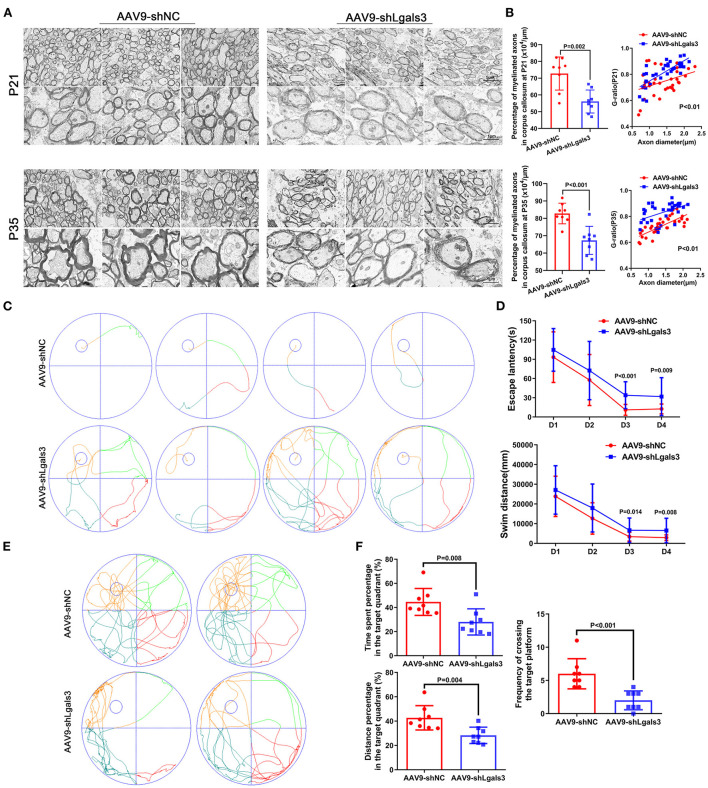
Gal-3 inhibition by AAV9-shLgals3 injection causes hypomyelination and causes cognitive deficits. Representative electron microscopy images of corpus callosum at P21 and P35 **(A)**. Quantification of myelinated axons percentage and G-ratio at P21 and P35 **(B)**, *N* = 8. Representative performance at day 4 of navigation test and its quantification **(C)**, quantification of escape latency and swim distance of navigation test **(D)**, representative performance at probe trail **(E)**, quantification of time spent and distance percentage in the target quadrant. Frequency of crossing the target platform **(F)**, *N* = 8.

We performed electron microscopy (EM) of corpus callosum to characterize myelin formation at P21 and P35 and displayed representative images in Figure 2A. Quantitative analyses showed increased G-ratios and decreased percentage of myelinated axons in AAV9-shLgals3 group at P21 and P35 (Figure 2B).

The spatial learning and memory were further assessed by the Morris water maze administered at 30 to 34 day after injection (P30-P34). Our results (Figures 2C,D) showed increased escape latency and swim distance in AAV9-shLgals3 group at day 3–4 of navigation test. Results of Figures 2E,F showed decreased platform crossing, time spent and distance percentage in the target quadrant in the probe trail.

### Recombinant Gal-3 administration attenuates cognitive deficits and drives remyelination after HI induced PWMI

Since Gal-3 knockdown impedes myelin formation, we wondered if exogenously rGal-3 administration will drive myelin formation during neurodevelopment. In order to investigate the efficacy of rGal-3 administration, we administrated right intracerebroventricular injection with rGal-3 (2 μl, 5 μg/ml, dissolved in sterile PBS, rGal-3 group) or sterile PBS (2 μl, Control group) at 10-day old of Sprague–Dawley rat pups.

However, our data demonstrated no significant difference on MBP and CNPase expression (SF 2A-C), mature oligodendrocyte number (SF 2D-E) between rGal-3 administration group (rGal-3) and PBS control group (Control) at 28 day old. In accordance with the former results, myelin formation accessed by EM at 30 day old of corpus callosum indicated no improvement after rGal-3 administration, further quantitative analyses demonstrated no significant difference between rGal-3 administration group and PBS control group on G-ratios and percentage of myelinated axons (SF 2F-G). These results indicate that rGal-3 administration show no benefit on myelin formation during neurodevelopment.

Previous works have confirmed a Gal-3 upregulation after CNS injury (Chip et al., 2017; Stajic et al., 2019; Al-Dalahmah et al., 2020), and Gal-3, which plays orchestrated roles in CNS, can be released by Iba-1 (+) microglia (Miron et al., 2013; Jiang et al., 2016; Ohtomo et al., 2018; Wang et al., 2019; Damisah et al., 2020). In this part, we intended to explore if rGal-3 administration is advantageous for remyelination after PWMI. Rats were divided into Control group, HI+PBS group, and HI+rGal-3 group, rats in HI+PBS group and HI+rGal-3 group underwent HI operation at 3 day old, and intracerebroventricular injection was performed with sterile PBS (2 μl, HI+PBS group) or rGal-3 (2 μl, 5 μg/ml, dissolved in sterile PBS, HI+rGal-3 group) at 7 days after injury (10-day old). No administration was performed in Control group.

**Figure 3:**
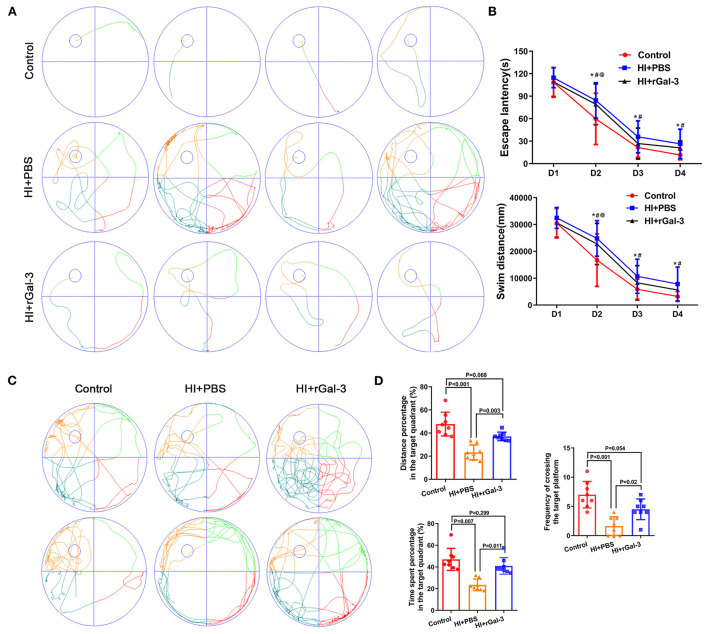
Recombinant Gal-3 administration attenuates spatial learning and memory deficits after HI induced white matter injury. Representative performance at day 4 of navigation test **(A)**. Quantification of escape latency and swim distance of navigation test **(B)**. *P < 0.05 (Control VS. HI+PBS), ^#^*P* < 0.05 (HI+PBS VS. HI+rGal-3), ^@^*P* < 0.05 (Control VS. HI+rGal-3), *N* = 8. Representative performance at probe trail **(C)**. Quantification of time spent and distance percentage in the target quadrant, frequency of crossing the target platform **(D)**, *N* = 8.

The spatial learning and memory were further characterized by the Morris water maze administered at 30 to 34 day after injury. Notably, our data indicated that rGal-3 administration significantly attenuated HI induced cognitive disorder, as manifested not only by decreased escape latency and swim distance at day3–4 of navigation test (Figures 3A,B), but also by increased platform crossing, time spent, and distance percentage traveled in the target quadrant in the probe trail (Figures 3C,D).

**Figure 4:**
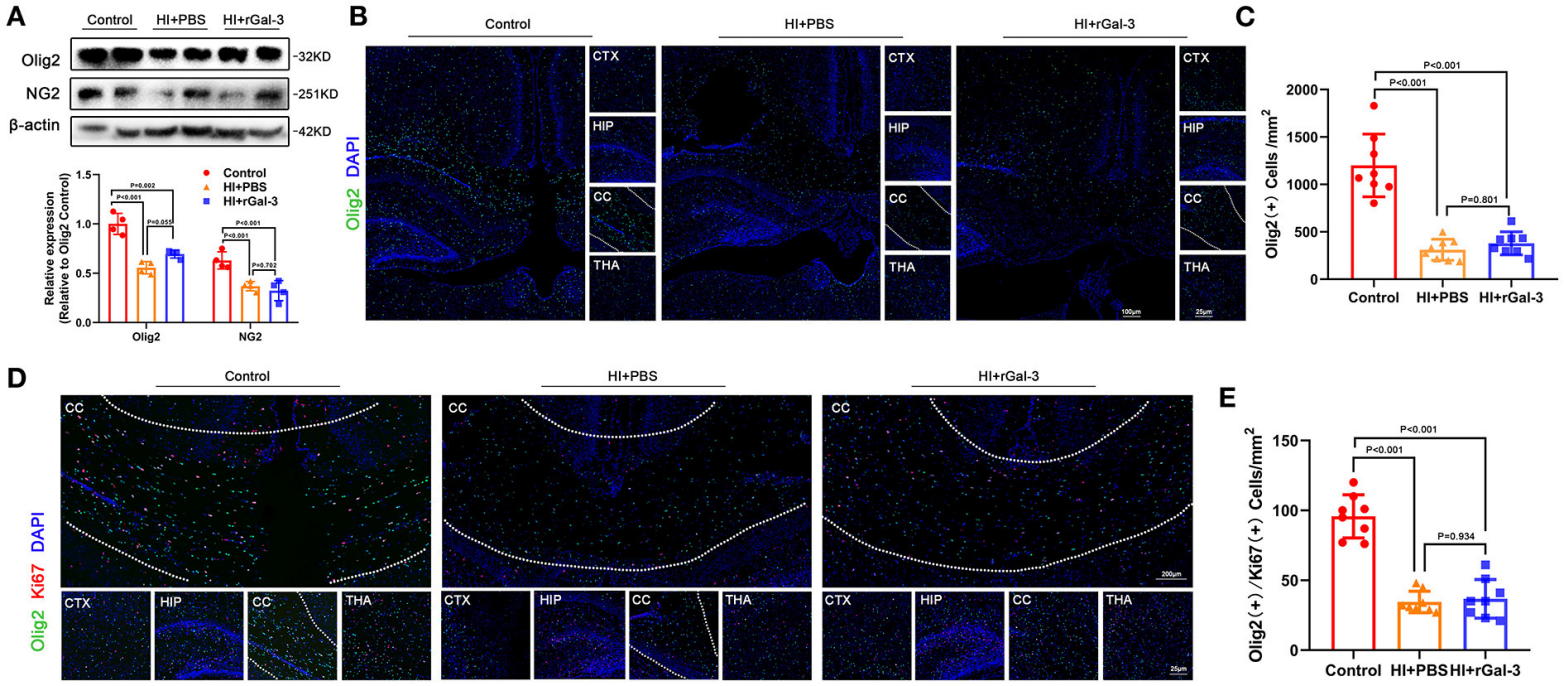
Recombinant Gal-3 administration does not accelerate proliferation of oligodendrocyte linage after HI induced white matter injury. Western blotting images and its quantification of Olig2 and NG2 at 14 day old **(A)**, *N* = 4. Representative immunofluorescence images of Olig2 (+) cells **(B)** and its quantification **(C)** at 14 day old. Representative double stained immunofluorescence images of Olig2 / Ki67 (+) cells **(D)** and its quantification **(E)** at 14 day old, *N* = 8.

Impressed with the extent of improvement on cognition, we performed western blotting of NG2 (a marker of early stage of oligodendrocyte linage) and Olig2, and immunofluorescence of Olig2 at 14 day after injury. Compared with Control group, rats in HI+rGal-3 and HI+PBS group showed decreased NG2 and Olig2 expression, Olig2 (+) cells number in corpus callosum at 14 day after injury, no significant difference was detected between HI+rGal-3 group and HI+PBS group (Figures 4A–C). Meanwhile, rats in HI+rGal-3 and HI+PBS group showed an Olig2 / Ki67 double-positive cells reduction than rats in Control group, and no significant difference was detected between HI+rGal-3 group and HI+PBS group (Figures 4D,E).

**Figure 5:**
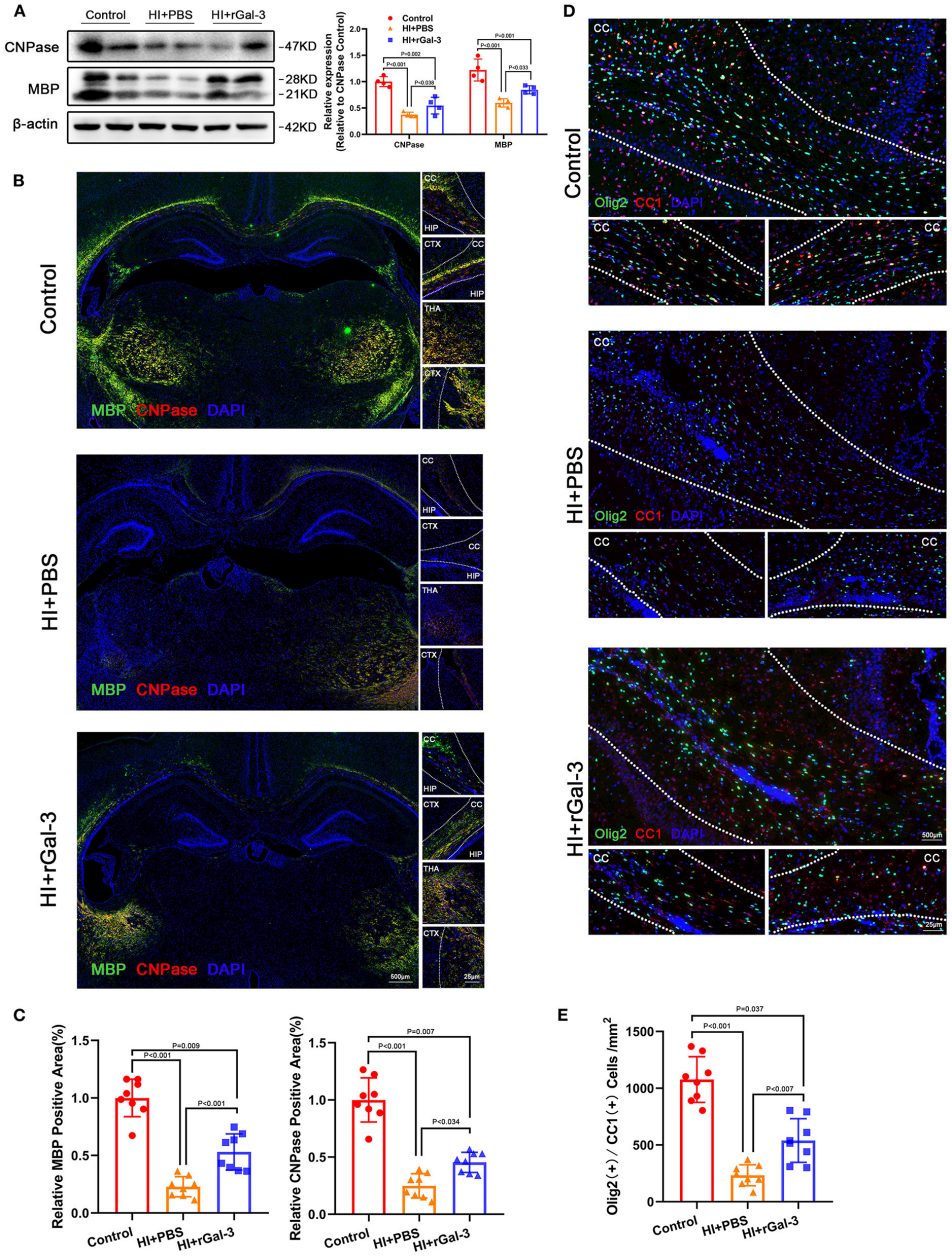
Recombinant Gal-3 administration increases mature oligodendrocytes number and oligodendroglial differentiation marker expression after HI induced white matter injury. Western blotting images and its quantification of MBP and CNPase at 28 day after HI injury **(A)**, *N* = 4. Representative immunofluorescence images **(B)** and its quantification **(C)** of MBP and CNPase at 28 day after HI injury, *N* = 8. Representative double stained immunofluorescence images **(D)** of Olig2 / CC1 (+) cells and its quantification **(E)** in the corpus callosum at P28, *N* = 8. CTX, Cortex; CC, Corpus callosum; HIP, Hippocampus; THA, Thalamus.

**Figure 6:**
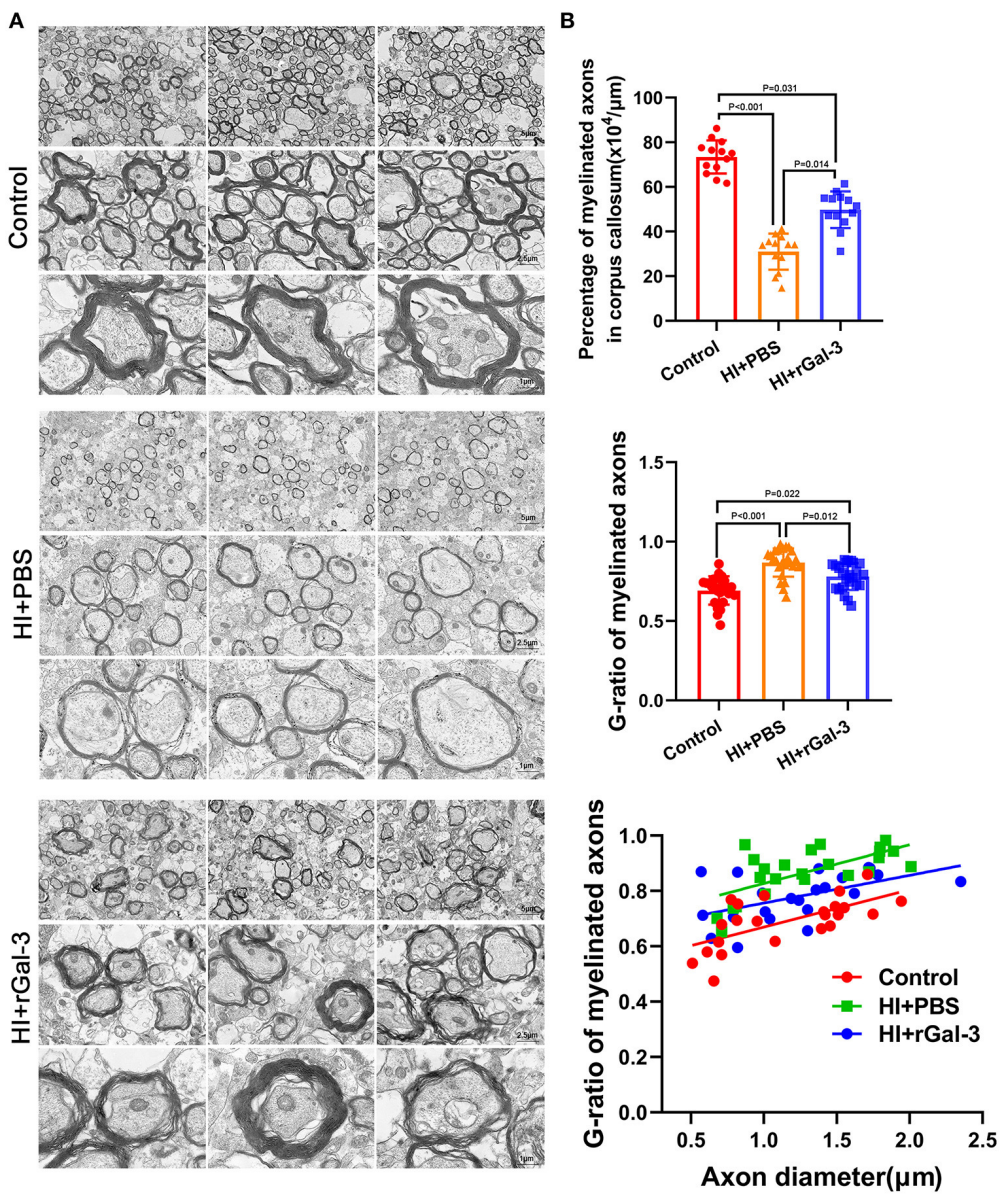
Recombinant Gal-3 administration drives remyelination after HI induced white matter injury. Representative electron microscopy images of corpus callosum at 35 day after HI injury **(A)**. Quantification of myelinated axons percentage and G-ratio at 35 day after HI injury **(B)**, *N* = 12.

Next, we performed western blotting and immunofluorescence of MBP and CNPase at 28 day after injury, we double stained Olig2 and CC1 at 28 day after injury. As expected, compared with Control group, rats in HI+PBS group showed decreased MBP and CNPase expression (Figures 5A–C), decreased Olig2 / CC1 (+) mature oligodendrocytes (Figures 5D,E), indicating myelin disruption after HI induced PWMI. Importantly, our data showed increased MBP and CNPase expression (Figures 5A–C), and Olig2 / CC1 (+) mature oligodendrocytes (Figures 5D,E) in HI+rGal-3 group than HI+PBS group. In addition, the effects of rGal-3 administration were further supported by EM at 35 day after injury, which revealed significant increased percentage of myelinated axons and a significant decreased G-ratios in the quantitative analyses (Figures 6A,B).

### Recombinant Gal-3 administration attenuates tissue injury and drives microglia toward anti-inflammatory phenotype activation

**Figure 7:**
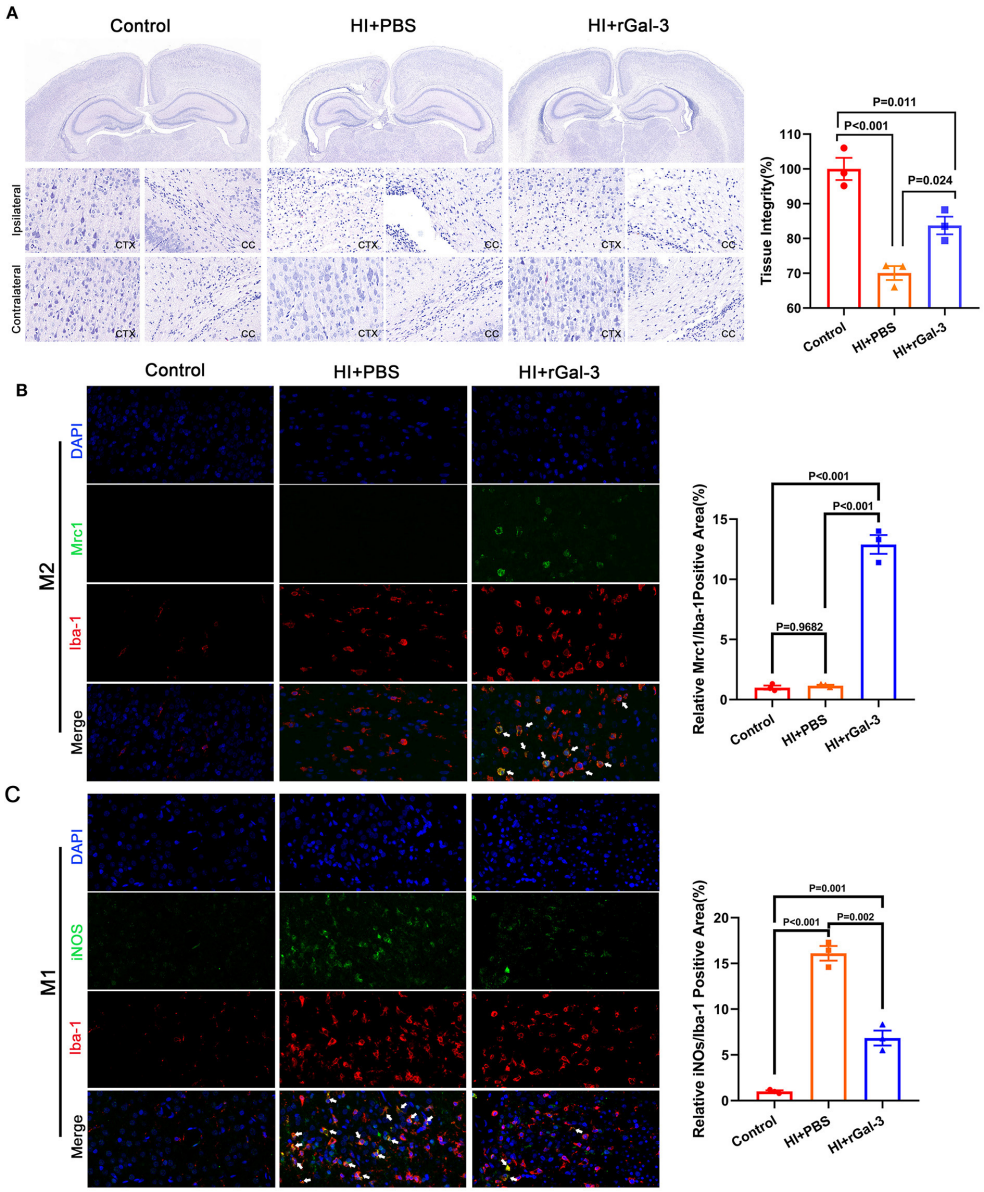
Recombinant Gal-3 administration attenuates tissue injury and drives microglia toward anti-inflammatory phenotype activation. Representative H-E staining images and its quantification at 28 day after HI injury **(A)**, *N* = 3. Representative immunofluorescence images and its quantification of Iba-1/ Mrc1 (+) microglia **(B)** and Iba-1/ iNOS (+) microglia **(C)** in corpus callosum at 14 day after HI injury, *N* = 3.

To gain further insight into the beneficial role of rGal-3 administration, we performed H-E staining to evaluate tissue injury, data was presented in Figures 7A,B, severe disorganized tissue, declined cell density and vacuoles were observed in the cortex and corpus callosum area in HI+PBS group, and rats in HI+rGal-3 group shown better tissue integrity after Gal-3 administration. Meanwhile, we double stained a microglia marker (Iba-1) with a M2 microglia marker (Mrc1), and Iba-1 with a M1 microglia marker (iNOS). Our data showed enhanced Iba-1/ Mrc1 (+) microglia and decreased Iba-1/ iNOS (+) microglia (Figures 7C,D), which indicated that rGal-3 administration favors microglia into an anti-inflammatory phenotype.

## Discussion

White matter injury is characterized by oligodendroglial death and myelin disruption. Perinatal hypoxic-ischemic induced white matter injury occurs one to eight per 1,000 live births in developed countries, which causes long-term neurological disability (Back, 2017; Ohtomo et al., 2018; Elbaz and Popko, 2019; Wu et al., 2019; Hughes and Appel, 2020; Willis et al., 2020). The importance of Gal-3 under normal condition and pathological condition has been illustrated previously (Hoyos et al., 2014; Chip et al., 2017; Dong et al., 2018; Kariya et al., 2018). Gal-3 knockdown not only downregulates trophic factors such as NGF and BDNF, but also causes gliogenesis disorder (Stajic et al., 2019; Al-Dalahmah et al., 2020). Meanwhile, Gal-3 knockout enlarges ischemic lesion and increases apoptotic neuron after stroke (Lalancette-Hébert et al., 2012). In order to identify the association between Gal-3 and myelin formation, we performed Gal-3 knockdown by AAV9-shLgals3 injection of Sprague-Dawley rats at 10 days old. In this study, in addition to increased mortality and developmental retardation, our data highlights the importance of Gal-3 on myelin formation. The efficacy of Gal-3 on myelin formation may contribute to (1) Gal-3 deletion was association with downregulation of nerve growth factor (NGF) and brain derived neurotrophic factor (BDNF) (Lalancette-Hébert et al., 2012; Stajic et al., 2019), which played crucial roles on neurodevelopment and myelin formation. (2) Gal-3 accelerates cell differentiation, and regulates cell-cell interactions within the extracellular matrix (Chip et al., 2017; Dong et al., 2018; Kariya et al., 2018), Gal-3 downregulation in oligodendrocyte linage may impede the ability of differentiation. (3) Microglia is able to phagocytose myelin sheaths and modify developmental myelination (Hughes and Appel, 2020), Gal-3 has been testified controlling microglial phagocytosis (Reichert and Rotshenker, 2019).

Impressed with the importance of Gal-3 during neurodevelopment, we wonder if upregulated Gal-3 accelerates myelin formation. Thus, we performed rGal-3 administration by intracerebroventricular injection. Although our data show no beneficial role of rGal-3 administration on myelin formation during neurodevelopment, we found rGal-3 administration attenuates cognitive deficits and drives remyelination after HI induced PWMI. Oligodendrocytes receive support from the environment and other type of glial cell such as microglia during neurodevelopment (Fulmer et al., 2014; Elbaz and Popko, 2019; Hughes and Appel, 2020; Willis et al., 2020). Microglia, acting as the principal immune cell of CNS, detects and responds to neuronal activity, engulfs surplus neurons and synapses, and selectively phagocytoses myelin sheaths to sculpt myelin formation in an activity-regulated manner (Hughes and Appel, 2020). A recent single-cell RNA-sequencing analysis of rodent confirmed *Mbp* mRNA expression within white matter microglia, which reveals the association between microglia and myelin formation under normal physiological conditions (Li et al., 2019). Meanwhile, microglia depletion experiments also reveal the essential role for postnatal proper development and homeostasis of oligodendrocytes and their progenitors (Hagemeyer et al., 2017). Microglia is able to react to modifications in the cellular environment through a graded response termed activation (Lalancette-Hébert et al., 2012). Activated microglia are highly heterogeneous with opposite activation statuses playing orchestrated role (Ohtomo et al., 2018; Damisah et al., 2020), which are pro-inflammatory phenotype (M1 microglia) and anti-inflammatory phenotype (M2 microglia) (Miron et al., 2013; Zabala et al., 2018). M1 microglia exists in all types of lesions and correlates with axonal damage, M1 microglia activation represents the initial steps of demyelinated lesion, accelerates infiltration of blood derived cells, and contributes to neurodegeneration lesions (Ramos-Cejudo et al., 2015; Ohtomo et al., 2018; Zabala et al., 2018; Hughes and Appel, 2020). Meanwhile, M2 microglia has been regarded as the anti-inflammatory or immunoregulatory functional phenotype, which drives oligodendrocyte differentiation during neurodevelopment, mediates tissue remodeling and wound healing, and counteracts pathological processes by releasing anti-inflammatory cytokines and growth factors (Miron et al., 2013; Ohtomo et al., 2018; Quenum Zangbede et al., 2018; Zabala et al., 2018).

Demyelinated lesions caused by PWMI are triggered by immune cell infiltration across the blood–brain barrier (BBB), which promotes inflammation, demyelination, and neuroaxonal degeneration (Ohtomo et al., 2018; Zabala et al., 2018; Elbaz and Popko, 2019; Willis et al., 2020). Previous studies have identified Gal-3 upregulation after CNS injury, and Gal-3 is released by several types of cells including microglia (Lalancette-Hébert et al., 2012; Hagemeyer et al., 2017; Quenum Zangbede et al., 2018; Stajic et al., 2019; Wang et al., 2019). Lalancette-Hebert et al. provide evidences that Gal-3 is interrelated with microglia proliferation and defective early activation (Lalancette-Hébert et al., 2012). Gal-3 is abundantly expressed in M2 microglia, and Gal-3 KD M2 microglia exhibits a deficient neutrophil clearance *in vitro* (Quenum Zangbede et al., 2018). Researchers have performed M2 microglia transfer from Gal-3 sufficient WT mice and found reduced neutrophilia in CNS with ameliorated disease severity in Gal-3 KO mice (Quenum Zangbede et al., 2018). Thus, we wondered if rGal-3 administration attenuates pro-inflammatory responses and selectively drives activated microglia toward M2 phenotype. In this study, we evaluated the expression of specific markers, and our results showed enhanced Iba-1/ Mrc1 (+) and decreased Iba-1/ iNOS (+).

Altogether, in this study, we knockdown Gal-3 to emphasize the significance on myelin formation. Then, we performed rGal-3 administration and provided evidence that rGal-3 attenuates cognitive deficits and drives remyelination after PWMI, which were accompanied with M2 microglia activation. Since myelin debris accumulation could be the result of demyelination or myelin phagotrophic defect, and phagocytosis of myelin debris is essential for efficient regenerative response and remyelination, which are modulated by M2 microglia activation (Miron et al., 2013; Ramos-Cejudo et al., 2015). We believe that rGal-3 administration turns on the switch and activates M2-associated functions after PWMI, which subsequently facilitates the clearance of infiltrating immune cells and myelin debris to improve remyelination. In summary, our work highlights the importance of Gal-3 on myelin formation and provide evidences that rGal-3 administration drives remyelination after HI induced PWMI.

## Data availability statement

The datasets used and/or analyzed during the current study are available from the corresponding author on reasonable request.

## Ethics statement

The animal study was reviewed and approved by the Animal Ethics Committee of Fudan University.

## Author contributions

QW, SD, HQ, and CC conceptualized the study. QW, SD, HQ, RG, MW, and QC performed postnatal hypoxic-ischemic brain injury operation and experimental operation. QW and CC drafted the manuscript. CC, ZL, and MX supervised the progress of the study and critically appraised the manuscript. All authors contributed to the article and approved the submitted version.

## Funding

This work was supported by National Key R&D Program of China (Grant number 2017YFA0104200), National Natural Science Foundation of China (Grant number 82101809), Basic and Applied Basic Research Project of Guangzhou (Grant number 202201011047), and 2020 Clinical PhD Starting Research Fund of Women and Children's Medical Center (Grant number 1600081).

## Conflict of interest

The authors declare that the research was conducted in the absence of any commercial or financial relationships that could be construed as a potential conflict of interest.

## Publisher's note

All claims expressed in this article are solely those of the authors and do not necessarily represent those of their affiliated organizations, or those of the publisher, the editors and the reviewers. Any product that may be evaluated in this article, or claim that may be made by its manufacturer, is not guaranteed or endorsed by the publisher.

## Supplementary material

Supplementary Figure 1 Gal-3 knockdown causes developmental retardation. **(A)** Western blotting images of Gal-3 and its quantification at P10, *N* = 3. **(B)** Body weight at the day of P0 [*N* = 49 (AAV9-shLgals3), *N* = 38 (AAV9-shNC)], P10 [*N* = 43 (AAV9-shLgals3), *N* = 38 (AAV9-shNC)], P14 [*N* = 37 (AAV9-shLgals3), *N* = 35 (AAV9-shNC)], P21 [*N* = 27 (AAV9-shLgals3), *N* = 27 (AAV9-shNC)], P28 [*N* = 16 (AAV9-shLgals3), *N* = 16 (AAV9-shNC)] and P35 [*N* = 8 (AAV9-shLgals3), *N* = 8 (AAV9-shNC)]. A survival curve **(C)** and mortality of AAV9-shLgals3 group and AAV9-shNC group from P0-P35 **(D)**. Representative immunofluorescence images of Olig2 (+) cells **(E)**, double stained immunofluorescence of Olig2/ Ki67 (+) cells **(F)** and quantification **(G)** at P21, *N* = 3. Representative H-E staining images of AAV9-shLgals3 and AAV9-shNC group at P28(H).

Supplementary Figure 2 Recombinant Gal-3 administration does not accelerate OPCs differentiation during neurodevelopment. Western blotting images and its quantification of MBP and CNPase at 28 day old **(A)**, *N* = 3. Representative immunofluorescence images of MBP and CNPase **(B)** and its quantification **(C)** at 28 day old, *N* = 8. Representative double stained immunofluorescence images of Olig2 / CC1 (+) cells **(D)** and its quantification **(E)** in the corpus callosum at 28 day old, *N* = 8. Representative electron microscopy images of corpus callosum at 30 day old **(F)**. Quantification of myelinated axons percentage and G-ratio at 30 day old **(G)**, *N* = 12.

## Abbreviations

HI: Hypoxic-ischemic
PWMI: Perinatal white matter injury
Gal-3: Galectin-3
rGal-3: Recombinant Gal-3
CNS: Central nervous system
BDNF: Brain derived neurotrophic factor
AAV: Adeno-Associated Virus
EM: Electron microscopy.
