# Supplementary figures and images for: Galectin-3 administration drives remyelination after hypoxic-ischemic induced perinatal white matter injury
Source: Front Cell Neurosci. 2022 Sep 20;16:976002. doi: 10.3389/fncel.2022.976002 (PMC9532057; doi:10.3389/fncel.2022.976002)

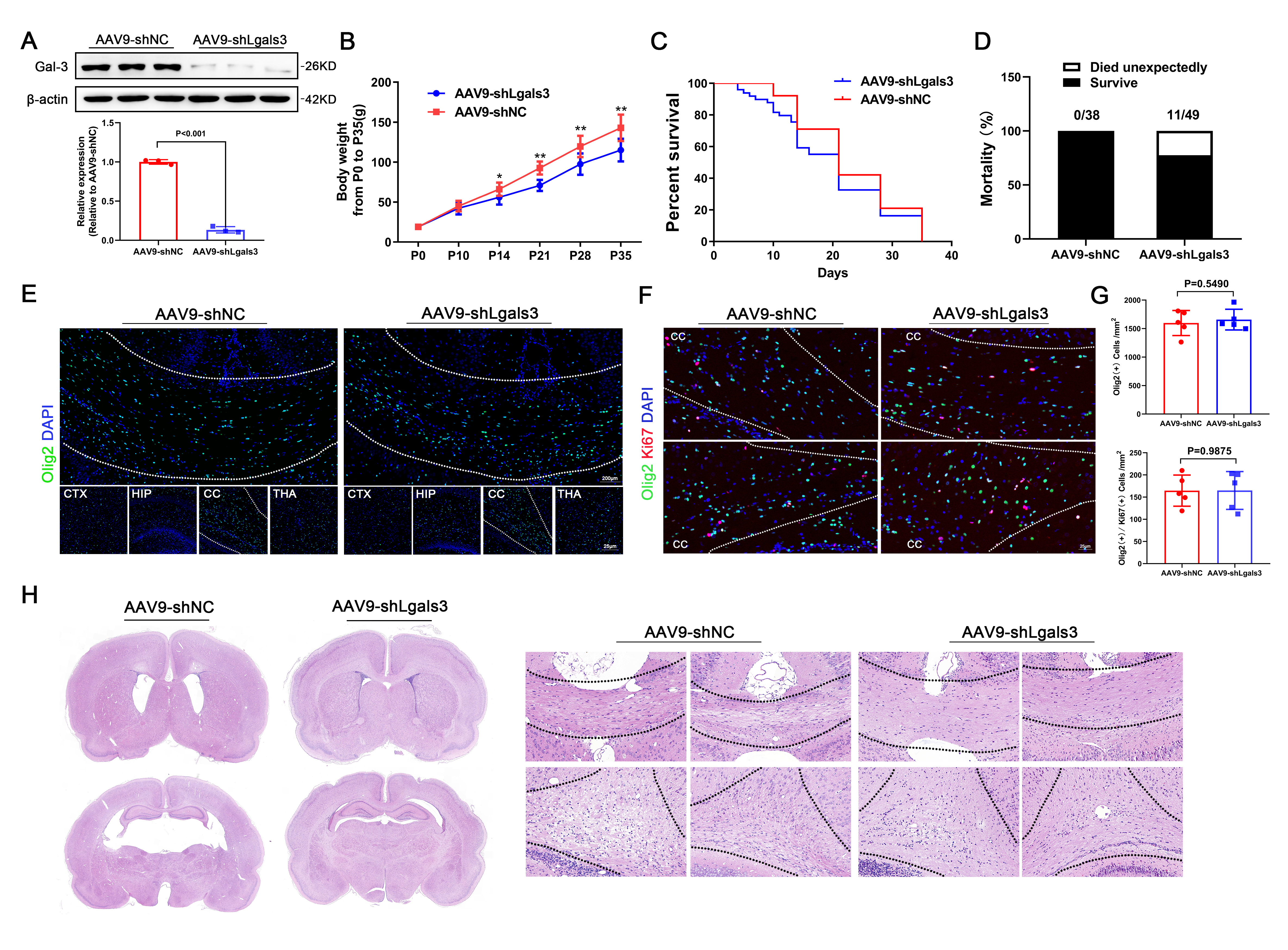

Supplement: Supplementary Figure 1 — Gal-3 knockdown causes developmental retardation. (A) Western blotting images of Gal-3 and its quantification at P10, N = 3. (B) Body weight at the day of P0 [N = 49 (AAV9-shLgals3), N = 38 (AAV9-shNC)], P10 [N = 43 (AAV9-shLgals3), N = 38 (AAV9-shNC)], P14 [N = 37 (AAV9-shLgals3), N = 35 (AAV9-shNC)], P21 [N = 27 (AAV9-shLgals3), N = 27 (AAV9-shNC)], P28 [N = 16 (AAV9-shLgals3), N = 16 (AAV9-shNC)] and P35 [N = 8 (AAV9-shLgals3), N = 8 (AAV9-shNC)]. A survival curve (C) and mortality of AAV9-shLgals3 group and AAV9-shNC group from P0-P35 (D). Representative immunofluorescence images of Olig2 (+) cells (E), double stained immunofluorescence of Olig2/ Ki67 (+) cells (F) and quantification (G) at P21, N = 3. Representative H-E staining images of AAV9-shLgals3 and AAV9-shNC group at P28(H). [file Image_1.TIF]

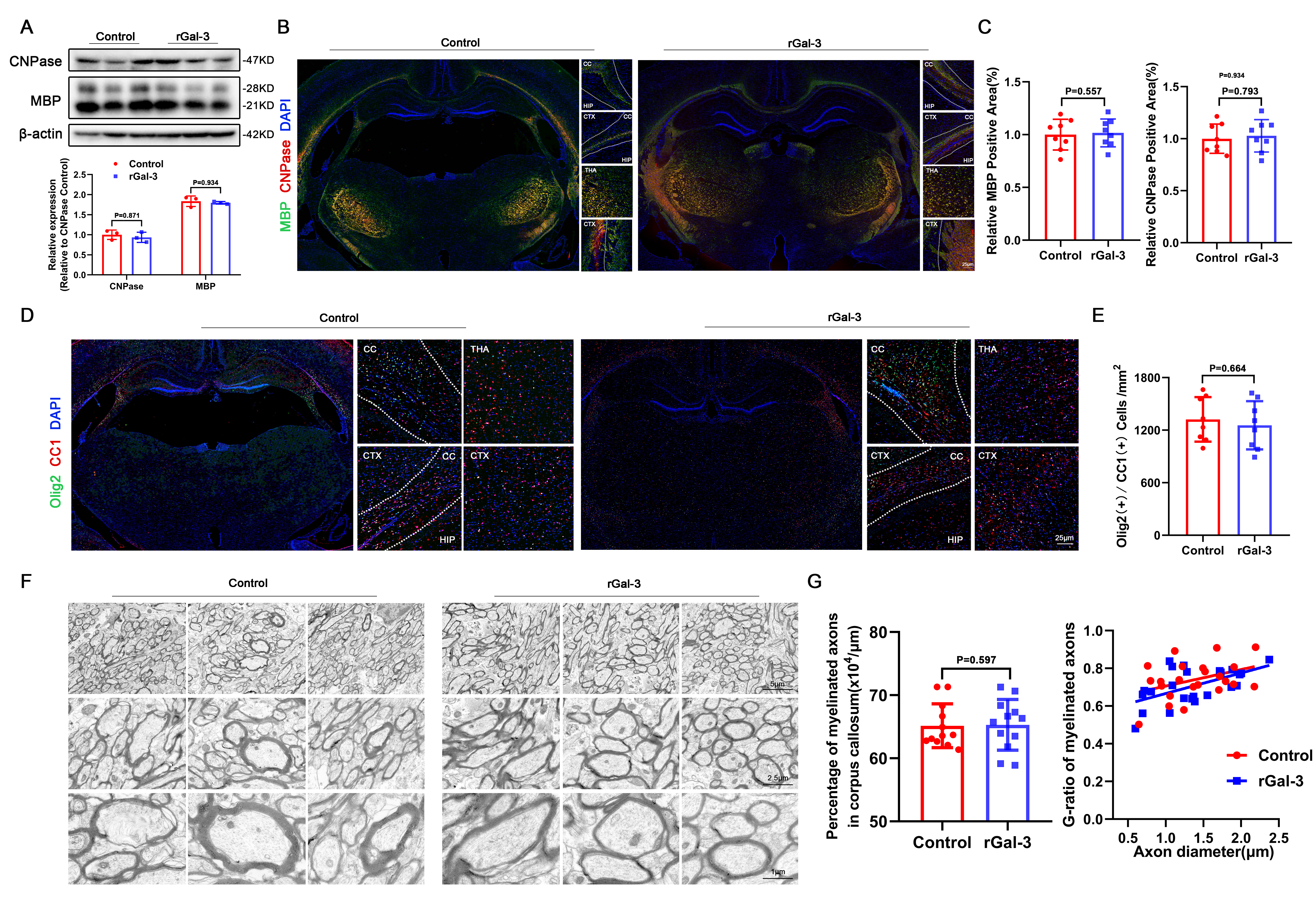

Supplement: Supplementary Figure 2 — Recombinant Gal-3 administration does not accelerate OPCs differentiation during neurodevelopment. Western blotting images and its quantification of MBP and CNPase at 28 day old (A), N = 3. Representative immunofluorescence images of MBP and CNPase (B) and its quantification (C) at 28 day old, N = 8. Representative double stained immunofluorescence images of Olig2 / CC1 (+) cells (D) and its quantification (E) in the corpus callosum at 28 day old, N = 8. Representative electron microscopy images of corpus callosum at 30 day old (F). Quantification of myelinated axons percentage and G-ratio at 30 day old (G), N = 12. [file Image_2.TIF]
